# Indoor air quality in urban and rural kindergartens: short-term studies in Silesia, Poland

**DOI:** 10.1007/s11869-017-0505-9

**Published:** 2017-08-17

**Authors:** Ewa Błaszczyk, Wioletta Rogula-Kozłowska, Krzysztof Klejnowski, Piotr Kubiesa, Izabela Fulara, Danuta Mielżyńska-Švach

**Affiliations:** 1 Environmental Toxicology Group, Institute for Ecology of Industrial Areas, 6, Kossutha St., 40-844 Katowice, Poland; 2 Department of Air Protection, Institute of Environmental Engineering, Polish Academy of Science, 34, Skłodowskiej-Curie St., 41-819 Zabrze, Poland; 3 Central Laboratory, Institute for Ecology of Industrial Areas, 6, Kossutha St., 40-844 Katowice, Poland; 4 Nursing Institute, Witold Pilecki State School of Higher Education, 8, Kolbego St., 32-600 Oświęcim, Poland

**Keywords:** Indoor air quality, Kindergarten, PM2.5, PAHs, *Salmonella* assay, Indoor/outdoor ratio

## Abstract

More than 80% of people living in urban areas who monitor air pollution are exposed to air quality levels that exceed limits defined by the World Health Organization (WHO). Although all regions of the world are affected, populations in low-income cities are the most impacted. According to average annual levels of fine particulate matter (PM2.5, ambient particles with aerodynamic diameter of 2.5 μm or less) presented in the urban air quality database issued by WHO in 2016, as many as 33 Polish cities are among the 50 most polluted cities in the European Union (EU), with Silesian cities topping the list. The aim of this study was to characterize the indoor air quality in Silesian kindergartens based on the concentrations of gaseous compounds (SO_2_, NO_2_), PM2.5, and the sum of 15 PM2.5-bound polycyclic aromatic hydrocarbons (PAHs), including PM2.5-bound benzo(a)pyrene (BaP), as well as the mutagenic activity of PM2.5 organic extracts in *Salmonella* assay (strains: TA98, YG1024). The assessment of the indoor air quality was performed taking into consideration the pollution of the atmospheric air (outdoor). I/O ratios (indoor/outdoor concentration) for each investigated parameter were also calculated. Twenty-four-hour samples of PM2.5, SO_2_, and NO_2_ were collected during spring in two sites in southern Poland (Silesia), representing urban and rural areas. Indoor samples were taken in naturally ventilated kindergartens. At the same time, in the vicinity of the kindergarten buildings, the collection of outdoor samples of PM2.5, SO_2_, and NO_2_ was carried out. The content of BaP and the sum of 15 studied PAHs was determined in each 24-h sample of PM2.5 (indoor and outdoor). In the urban site, statistically lower concentrations of SO_2_ and NO_2_ were detected indoors compared to outdoors, whereas in the rural site, such a relationship was observed only for NO_2_. No statistically significant differences in the concentrations of PM2.5, PM2.5-bound BaP, and Σ15 PAHs in kindergartens (indoor) versus atmospheric (outdoor) air in the two studied areas were identified. Mutagenic effect of indoor PM2.5 samples was twice as low as in outdoor samples. The I/O ratios indicated that all studied air pollutants in the urban kindergarten originated from the ambient air. In the rural site concentrations of SO_2_, PM2.5 and BaP in the kindergarten were influenced by internal sources (gas and coal stoves).

**Electronic supplementary material:**

The online version of this article (doi:10.1007/s11869-017-0505-9) contains supplementary material, which is available to authorized users.

## Introduction

According to the World Health Organization (WHO) atmospheric air (outdoor) pollution contributed to 3 million premature deaths worldwide per year in 2012 due to exposure to particulate matter (PM)10 (fraction of atmospheric particles with aerodynamic diameter not greater than 10), which causes cardiovascular and respiratory disease, and cancers (WHO 2016). Many epidemiological studies report that short-term and long-term exposures to atmospheric particulate matter, especially its fine fraction (particles with diameter of 2.5 μm or less, PM2.5), are responsible for many harmful effects on human health (Brunekreef and Holgate 2002; Kim et al. 2011). Nevertheless, it is not only the size of the atmospheric particles that has a remarkable influence on the harmful health effects of PM2.5 but also its chemical composition. A group of organic pollutants, which may be components of the PM, are polycyclic aromatic hydrocarbons (PAHs). PAHs are formed by the incomplete combustion of organic materials, and they are constituted by carbon and hydrogen arranged in two or more fused aromatic rings. Based on their carcinogenic and mutagenic potential and wide distribution in the environment, 16 PAHs have been included on the list of priority pollutants issued by the Environmental Protection Agency of the USA, US EPA (Lerda 2011). In atmospheric conditions, the most carcinogenic PAHs, like benzo(a)pyrene and dibenz(a,h)anthracene, are mainly bound to the PM, particularly to the PM2.5 (Li et al. 2009; Rogula-Kozłowska 2016).

A commonly identified congener of PAHs in the atmospheric air is benzo(a)pyrene (BaP), which in 2012 was classified to the highly genotoxic compounds. According to the International Agency for Research on Cancer (IARC), it belongs to group 1—carcinogenic to humans (IARC 2012). Moreover, some products containing BaP, e.g., tobacco smoke, exhaust from coal combustion, diesel exhaust, are also classified as carcinogens (IARC 2016). It is known that BaP and some other PAHs induce cancer by a mutagenic mechanism that involves metabolic activation to reactive diol-epoxides that covalently bind to DNA (Błaszczyk and Mielżyńska-Švach 2017). The health evaluation data suggest that lung cancer is the most serious health risk from exposure to PAHs, not only in the atmospheric air but also in the indoor air (WHO 2005, 2010). Some epidemiological studies supported a possible correlation between lung cancer and the mutagenic activity of the organic matter in the outdoor air of urban areas (Buschini et al. 2001; Zhao et al. 2003; Gilli et al. 2007). In order to evaluate the mutagenic activity of airborne particulate matter, the *Salmonella*/microsome assay was applied, which allowed detecting a broad range of chemical substances causing genetic damage (Mortelmans and Zeiger 2000; Claxton et al. 2004; Brito et al. 2013).

People spend most of their time in indoor environments (88.9%), with limited time spent outdoors (5.8%) or in vehicles (5.36%) (Matz et al. 2014), and as a consequence, they may be exposed to many pollutants of indoor origin, particularly fine PM and its subfraction—ultrafine particles, which may cause cardiovascular, respiratory, and neurological hazards to human health (Diffey 2011). Morawska et al. (2013) reported that 19–76% of the integrated daily residential exposure to ultrafine particles originated from indoor-generated particles. Study of the PAH characteristics in indoor/outdoor PM10, PM2.5, and PM1, carried out by Hassanvand et al. (2015) in a retirement home and a school dormitory in Teheran, indicated that the concentration of PAHs bound to PM was predominantly (83–88%) found in the PM2.5 fraction, which can penetrate deeper into the alveolar regions of the lungs.

Children spend most of their time in indoor environments, and therefore, are more exposed to pollution indoors than outdoors. They are particularly vulnerable to the harmful effects of air pollution, because they breath relatively higher volumes of air in relation to their body weights, have immature lung defenses, narrower airways, higher inhalation rates, and metabolic rate of oxygen consumption per unit of body weight; furthermore, their tissue and organs are growing (Mendell and Heath 2005; Salvi 2007; Branco et al. 2014). The exposure of children to air pollution can result in asthma and other respiratory symptoms, even in the case of low exposure (White et al. 2009; Hulin et al. 2010; Gül et al. 2011; Kim et al. 2011; Buonanno et al. 2012).

During the past decade, many studies were conducted to assess indoor air quality, mainly in school environments. A large number of indoor air pollutants were measured, including sulfur dioxide (SO_2_), nitrogen oxides (NO_x_), ozone (O_3_), carbon mon- and dioxide (CO and CO_2_), volatile organic compounds (VOCs), bioaerosols, PM, and PAHs, and selected heavy metals, including mercury (Mendell and Heath 2005; Stranger et al. 2008; Demirel et al. 2014; Rivas et al. 2014; Gatto et al. 2014; Mainka and Zajusz-Zubek 2015; Tofful and Perrino 2015; Majewski et al. 2016), but very few authors reported results on indoor air quality in kindergartens or day care centers (Wichmann et al. 2010; Branco et al. 2014; Mainka and Zajusz-Zubek 2015). Mutagenicity of indoor particulate matter evaluated using TA98 and YG1024 strains was examined only in places of residence in Japan, Thailand, and China (Zhou et al. 2000; Takagi et al. 2002; Chunram et al. 2007). According to the expected development of early childhood education, particular attention should be paid to ensure high-quality care, and especially to the indoor environmental quality, which is crucial in relation to children’s health and welfare.

The aim of the study is to characterize indoor air quality in two kindergartens located in urban (Dąbrowa Górnicza) and rural (Złoty Potok) areas of Silesia, Poland, taking into consideration the air pollution by PM2.5, 15 PM2.5-bound PAHs, SO_2_, NO_2_, and the *Salmonella* mutagenicity of PM2.5. Influence of atmospheric pollution on air quality in kindergartens was assessed and discussed.

## Materials and methods

### Sampling sites and methods

The studied area was located in the southern part of Poland (Silesia). Indoor and outdoor samples of gaseous pollutants (SO_2_, NO_2_) and PM2.5 were collected in two sites during the spring season (17 March–09 April 2010 in Złoty Potok and 10 April–3 May 2010 in Dąbrowa Górnicza). The first site represents an urban-industrial area in Dąbrowa Górnicza, and the second one, a rural region in Złoty Potok. The sampling site in Dąbrowa Górnicza (geographic coordinates: latitude 50.329111° North, longitude 19.231222° East, elevation 281 m a.s.l.) may be considered as an urban background site according to Directive 2008/50/EC. The sampling site in Złoty Potok (geographic coordinates: latitude 50.710889° North, longitude 19.458797° East, elevation 291 m a.s.l.) represented a regional background site (Directive 2008/50/EC). The location of the described sampling points is presented in the Supplementary Material (SM1).

In both sites, the air samples were collected in parallel indoors and outdoors. Indoor air was sampled in two kindergartens. The first kindergarten in Dąbrowa Górnicza was located in a two-storied detached building with six classrooms, a locker room, and a kitchen. The kindergarten in Złoty Potok was smaller and situated in a single-storied detached building with one didactic room and one room where children used to play and eat meals. A detailed characteristic of both kindergartens is presented in the Supplementary Material (SM2). Outdoor air samples were collected in the vicinity of the kindergarten buildings. The sampling points in Dąbrowa Górnicza and Złoty Potok belong to the Regional Inspectorate of Environmental Protection (RIEP) in Katowice(http://www.katowice.pios.gov.pl/). Sequential Dichotomous Partisol-Plus (Model 2025) was used for outdoor 24-h PM2.5 sample collection (air volume 1 m^3^/h). The 24-h PM2.5 samples of indoor particles were taken with a PNS-15 sampler (Atmoservice LVS, air volume 2.3 m^3^/h). All samples of PM2.5 were collected on quartz fiber filters (QMA: 47 mm in diameters). Concentrations of PM2.5 samples were determined according to the reference method for gravimetric measurements (EN 12341:2014). More detailed descriptions of the localities and sampling procedures for PM2.5 as well as atmospheric conditions are published in Błaszczyk et al. (2017).

Indoor samples for determination of SO_2_ and NO_2_ concentrations were collected with diffusive samplers—IVL Swedish Environmental Research Institute Ltd., Gothenburg, Sweden. First, the suction air stream passed through a filter paper, and then it was split and directed to the scrubber, wherein the sulfur dioxide was absorbed, whereas nitrogen dioxide was absorbed on a sintered glass. The amount of air passing through the system was calculated using two glass capillaries mounted on the suction pump and was equal to 1 m^3^ per for the glass scrubber (SO_2_) and 0.5 m^3^ per 24 h for the glass frit (NO_2_). Volume of air was measured with a marked gas meter. The operation of the aspirator was based on a weekly exchange of sintered glass and solutions in sorbent scrubbers. The values of outdoor 24-h average concentrations of SO_2_ and NO_2_ in the two studied sites were taken from the urban and RIEP (http://www.katowice.pios.gov.pl/). In this study, in total forty 24-h samples for each compound (SO_2_, NO_2_, and PM2.5) were collected (twenty 24-h samples in Dąbrowa Górnicza and Złoty Potok, respectively). For each selected pollutant, in each location, ten air samples in kindergartens (indoor samples) and ten samples of atmospheric air (outdoor samples) were collected.

### SO_2_ and NO_2_ analysis

The sorption of SO_2_ takes place in 0.3% H_2_O_2_ solution. Sulfur dioxide was oxidized to sulfate ion, which was determined by ion chromatography using DX100 ion chromatograph (Dionex, USA) with AO4S column. Daily concentrations of SO_2_ absorbed in the glass scrubber were calculated (Svanberg et al. 1998).

Nitrogen dioxide absorbed on the sintered glass was reduced to nitrite ion in an aqueous-glycol medium containing iodide and arsenate ions. After exposure, the sintered glass was washed with water, and the nitrite ion concentration was determined by spectrophotometry (Specol 11) at a wavelength of 540 nm. Then, the daily concentration of NO_2_ absorbed by the sintered glass was calculated (Svanberg et al. 1998).

### PAHs analysis

The sum of 15 PM2.5-bound PAHs was determined based on the concentrations of the following compounds: naphthalene (NP), acenaphthene (ACE), fluorene (FL), phenanthrene (PHE), anthracene (ANT), fluoranthene (FLA), pyrene (PYR), benz(a)anthracene (BaA), chrysene (CHR), benzo(b)fluoranthene (BbF), benzo(k)fluoranthene (BkF), benzo(a)pyrene (BaP), dibenz(a,h)anthracene (DahA), benzo(g,h,i)perylene (BghiP), and indeno(1,2,3-c,d)pyrene (IcdP), which were identified according to the standard methodology (EN 15549:2008). Each sample of PM2.5 deposited on the filter was extracted in an accelerated solvent extractor (ASE 350, Dionex) with dichloromethane (DCM) as an extraction solvent. Analysis of the extract was carried out using high-performance liquid chromatography with a fluorescence detector (HPLC-FLD 1200, Agilent Technologies). PAH resolution was performed using RP C18 column (PAH LiChrospher 5 μm × 250 × 3 mm, Merck) and gradient elution: water-methanol. Qualitative and quantitative assessment of PAHs was made using a fluorescence detector (FLD), with time-programmable excitation and emission wavelength changes: for NP, ACE, and FL—220/330 nm; for PHE, ANT, FLA, PYR, BaA, and CHR—260/420 nm; for BbF, BkF, BaP, DahA, and BghiP—290/450 nm; and for IcdP—248/500 nm. A detailed description of the PAH analysis containing extraction and concentration procedure as well as calibration parameters and quality control description was presented in Błaszczyk et al. (2017).

### *Salmonella*/microsome assay

Dichloromethane extracts of PM2.5 samples, remaining after the determination of PAHs, were used to investigate the mutagenic activity. In addition, dichloromethane extracts from clean filters were prepared and used as a blank sample. They were stored at −20 °C unless all extracts were tested using the *Salmonella* assay. Dry aerosol extracts of PM2.5 were dissolved in dimethyl sulfoxide (DMSO) in a volume corresponding to the highest volume of the air passing through the filter.

The organic extracts were assayed for mutagenicity using the *Salmonella*/microsome assay (Maron and Ames 1983; Mortelmans and Zeiger 2000). *Salmonella typhimurium* TA98 (frameshift strain) and its derivative strain YG1024 (*O*-acetyltransferase overproducing), obtained from Prof. Takehiko Nohmi, the National Institute of Health in Tokyo, were used, with and without metabolic activation (± S9 fraction). The choice of the TA98 and YG1024 strains was based on the sensitivity of these strains to certain chemical compounds responsible for mutagenic effects caused by particulate matter. PAHs belong to chemical pollutants which can induce frameshift mutations rather than base-pair substitutions in bacterial strains of *Salmonella*. Results of the previously carried out studies indicate that in Poland during winter season, the most suitable strain used for testing PM mutagenicity is TA98 and its YG derivatives (YG1021, YG1024, YG1041) (Zwoździak et al. 2001; Bełcik et al. 2014). Relevant diagnostic tests of the tester strains were routinely carried out, including crystal violet, UV, and ampicillin and tetracycline sensitivities (Claxton et al. 2004).

Aroclor 1254-induced, Sprague–Dawley rat liver S9 was obtained from TRINOVA Biochem GmbH Germany. The S9-mix used in these assays consisted of (per ml) 0.6 ml of 0.2 M phosphate buffer (pH 7.4) containing 0.4 M MgCl_2_ and 1.65 M KCl, 0.1 ml of 4 M NADP, 0.1 ml of 5 μM glucose 6-phosphate, and 32 mg of S9 protein. The mixture was sterilized by filtering through a 0.45 μm filter prior to use in the assay.

The mutagenic effect of PM2.5 collected outdoor was tested at doses corresponding to 1, 2, and 4 m^3^ of air. In the case of indoor samples, doses corresponding to 2, 4, and 8 m^3^ were used. The tested doses were added to 2.5 ml of sterilized top agar (0.6% agar and 0.5% NaCl containing 0.5 mM of histidine and 0.5 mM of biotin) and poured onto minimal glucose agar plates [1 × Vogel-Bonner salts (0.2 g/l magnesium sulfate, 2 g/l citric acid monohydrate, 10 g/l dipotassium hydrogen phosphate, and 3.5 g/l sodium ammonium phosphate), 2% glucose, and 1.5% agar]. The plates were then incubated at + 37 °C for 48 h (TA98) and 72 h (YG1024), and then histidine-independent revertant colonies were counted using a colony counter. In parallel, the number of spontaneous revertants and the number of revertants after adding the blank samples with and without S9 fraction were analyzed. Apart from that, levels of revertants induced by direct and indirect mutagen were tested, which for the TA98 strain were nitrocholino-4-N-oxide (NQNO) and BaP, and for the strain YG1024: 1-nitropirene (1-NP) and 2-aminofluorene (2-AF).

### Statistical analysis

The obtained results were analyzed using the package Statistica for Windows, version 10.

**Fig. 1:**
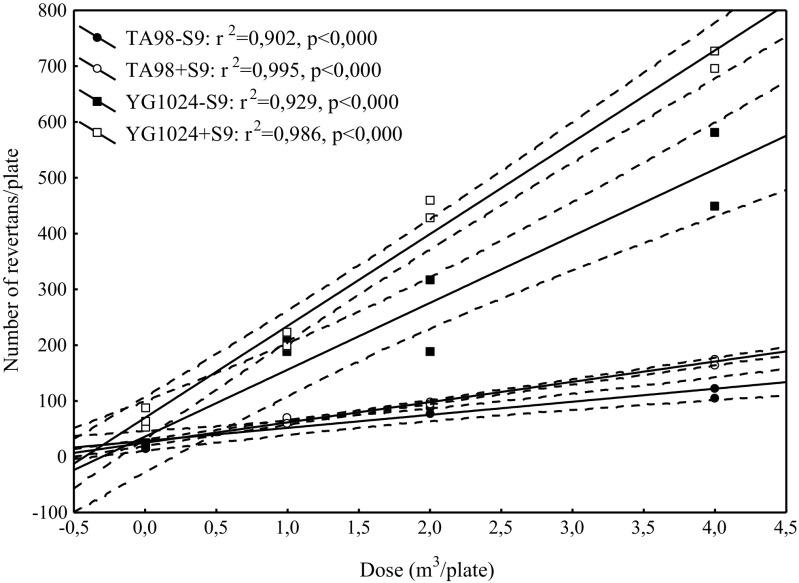
Dose-response relationships for outdoor PM2.5 fraction collected at air quality monitoring stations in Dąbrowa Górnicza

**Fig. 2:**
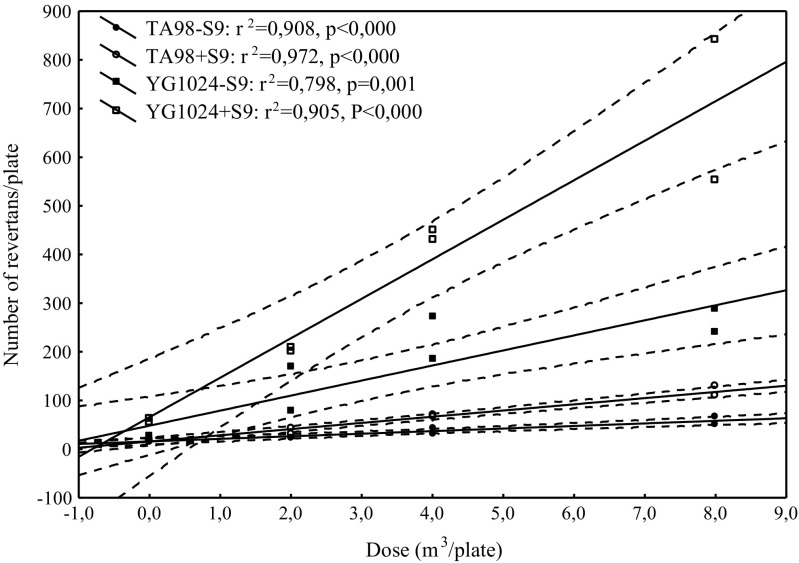
Dose-response relationships for indoor PM2.5 fraction in the kindergarten in Dąbrowa Górnicza

The mutagenic effect of PM2.5 organic extracts was calculated using the number of revertants as a dependent variable and dose of extract as an independent variable. To select the doses with linear dose-response relationship, a point-rejection method was applied. The assessment of data fitting to the model was made by the least squares method involving a regression analysis and analysis of variance instead of a maximum likelihood method proposed by Bernstein et al. (1982), with the assumption of a significant level p equal or lower than 0.05. Examples of dose-response relationship are shown in Figs. 1 and 2. Based on the parameters of this equation, the expected number of revertants induced by 1 m^3^ of the sampled air was computed (rev/m^3^). The sample was considered to be mutagenic when the number of revertants was twice as high as that of the corresponding negative control sample and when a significantly positive dose-response relationship was observed.

Summary statistics (mean, standard error, median, minimum, and maximum) was determined for each of the pollutants. Since the concentrations of the measured pollutants were not normally distributed, a nonparametric Mann-Whitney *U* test was used to compare indoor-outdoor differences, and indoor-outdoor correlations were described using Spearman’s correlation coefficients (rs). The I/O ratio of pollutant concentration was used to justify the presence of indoor sources (I/O > 1) or infiltration of the ambient air (I/O ≤ 1). Statistical significance was assumed at *p* ≤ 0.05.

## Results and discussion

### Gaseous pollutants

Guidelines of the European Parliament for atmospheric air quality (Directive 2004, 2008) and from WHO for indoor air quality (WHO 2010) are listed in Table 1. Table 2 summarizes the indoor and outdoor 24-h concentrations of SO_2_ and NO_2_ in Dąbrowa Górnicza and Złoty Potok.

**Table 1:** EU and WHO guidelines for ambient (outdoor) and indoor air quality (WHO 2005, 2010, 2016)

Pollutants	EU guidelines for ambient air quality	WHO guidelines for ambient air quality	WHO guidelines for indoor air quality
SO_2_	350 μg/m^3^ (1-h average) 125 μg/m^3^ (24-h average) 20 μg/m^3^ (annual average)	500 μg/m^3^ (10-min average) 20 μg/m^3^ (24-h average)	
NO_2_	200 μg/m^3^ (1-h average) 40 μg/m^3^ (annual average)	200 μg/m^3^ (1-h average) 40 μg/m^3^ (annual average)	200 μg/m^3^ (1-h average) 40 μg/m^3^ (annual average)
PM2.5	25 μg/m^3^ (annual average)	10 μg/m^3 3^ (annual average) 25 μg/m^3^ (24-h average)	
BaP	1 ng/m^3^ (annual average)		No safe threshold 1.2 ng/m^3^ (cancer risk of 10^4^) 0.12 ng/m^3^ (cancer risk of 10^5^) 0.012 ng/m^3^ (cancer risk of 10^9^)

**Table 2:** Indoor and outdoor air pollutants in kindergartens located in two investigated sites in Silesia, Poland

Pollutant	Indoor			Outdoor			*p* ^b^
	Mean ± SE	Median	Range	Mean ± SE	Median	Range	
Dąbrowa Górnicza							
SO_2_	8.6 ± 0.9	8.6	4.3–12.7	12.9 ± 0.9	13.5	9.0–16.0	0.005^c^
NO_2_	8.2 ± 0.3	8.1	6.8–9.8	35.1 ± 5.3	34.5	19.0–55.0	0.000^c^
PM2.5	28.2 ± 3.1	25.1	18.5–42.4	32.4 ± 02.7	32.5	22.8–45.0	0.259
BaP	3.7 ± 0.8	3.6	1.2–7.5	4.2 ± 0.8	4.0	1.1–8.0	0.456
Σ15 PAHs^a^	38.8 ± 7.1	36.1	14.6–72.9	57.3 ± 12.4	52.9	18.0–117.7	0.209
TA98 − S9	21.4 ± 0.3	21.2	21.0–21.9	47.3 ± 3.6	50.3	36.6–51.8	0.034^c^
TA98 + S9	27.9 ± 0.4	28.3	27.0–28.4	53.3 ± 7.4	56.8	33.1–66.6	0.034^c^
YG1024 − S9	63.1 ± 8.0	56.7	53.6–79.0	140.5 ± 17.6	146.7	93.1–175.5	0.034^c^
YG1024 + S9	100.3 ± 23.7	85.4	68.9–146.7	186.4 ± 32.2	192.6	114.0–246.4	0.077
Złoty Potok							
SO_2_	10.1 ± 0.6	10.4	6.3–12.3	10.0 ± 1.7	9.0	4.0–16.0	0.881
NO_2_	8.2 ± 1.2	7.8	4.2–13.5	10.5 ± 1.0	10.0	8.0–17.0	0.016^c^
PM2.5	31.9 ± 3.3	36.1	20.0–41.9	32.8 ± 3.0	30.6	16.0–69.5	0.939
BaP	5.7 ± 1.59	3.1	2.7–12.8	3.6 ± 0.6	3.1	1.0–8.1	0.106
Σ15 PAHs^a^	45.35 ± 9.4	31.4	25.0–89.5	51.2 ± 8.5	39.3	22.8–107.8	0.704
TA98 − S9	20.4 ± 1.4	19.6	18.6–23.1	43.1 ± 6.1	39.5	32.6–60.8	0.010^c^
TA98 + S9	28.7 ± 0.9	27.8	27.8–30.4	46.7 ± 6.3	45.4	33.5–62.7	0.014^c^
YG1024 − S9	58.6 ± 2.9	58.4	53.7–63.8	134.4 ± 24.5	121.9	89.6–204.2	0.019^c^
YG1024 + S9	75.5 ± 2.4	73.2	72.9–80.4	169.3 ± 35.8	150.6	105.2–270.9	0.019^c^

Units: SO_2_, NO_2_, and PM2.5 (μg/m^3^), BaP and Σ15 PAHs (ng/m^3^), TA98 ± S9 and YG1024 ± S9 (rev/m^3^)

^a^Naphthalene, acenaphthene, fluorine, phenanthrene, anthracene, fluoranthene, pyrene, benz(a)anthracene, chrysene, benzo(b)fluoranthene, benzo(k)fluoranthene, benzo(a)pyrene, benzo(g,h,i)perylene, dibenz(a,h)anthracene, and indeno(1,2,3-c,d)pyrene

^b^Mann-Whitney test, differences between indoor vs outdoor air

^c^Statistically significant differences at the level *p* ≤ 0.05

### Sulfur dioxide

The indoor 24-h concentrations of SO_2_ ranged from 4.3 to 12.7 μg/m^3^ with the median value of 8.6 μg/m^3^ for Dąbrowa Górnicza and from 6.3 to 12.3 μg/m^3^ with the median of 10.4 μg/m^3^ for Złoty Potok. The average outdoor concentration of SO_2_ was 13.5 and 9.0 μg/m^3^ for Dąbrowa Górnicza and Złoty Potok, respectively; 24-h concentrations varied from 9.0–16.0 μg/m^3^ to 4.0–16.0 μg/m^3^ (Table 2). In Dąbrowa Górnicza, 24-h outdoor concentrations of SO_2_ were statistically higher (*p* = 0.004) than its indoor concentrations.

According to the European Directive, the limits for SO_2_ are established only for atmospheric air and are 125 μg/m^3^ for 24-h exposure (not to be exceeded more than three times in any calendar year) and 20 μg/m^3^ for annual exposure (Table 1). In Dąbrowa Górnicza and Złoty Potok, both 24-h and the average indoor and outdoor SO_2_ concentrations did not exceed the permissible levels. However, taking into consideration the short time of the measurement period, it cannot be excluded that the annual monitoring would not show the exceedances.

Studies concerning indoor SO_2_ are limited. For example, in Belgian primary schools during winter, the indoor and outdoor 24-h concentrations of SO_2_ in the urban area ranged from 1.9 to 2.9 μg/m^3^ (mean 2.4 μg/m^3^) and from 2.8 to 10.2 μg/m^3^ (mean 5.9 μg/m^3^), respectively, whereas in the suburban area, from 1.4 to 3.5 μg/m^3^ (mean 2.4 μg/m^3^) and from 4.2 to 15.4 μg/m^3^ (mean 7.7 μg/m^3^), respectively (Stranger et al. 2008). These concentrations were much lower than the levels of SO_2_ in Dąbrowa Górnicza and Złoty Potok. Lower concentrations of indoor SO_2_ were also found in two Turkish schools—5.6 μg/m^3^ (passive sampling) and 6.7 μg/m^3^ (active sampling), whereas the outdoor concentration of this pollutant was definitely higher (39.6 μg/m^3^) than in our study (Bozkurt et al. 2015).

Most of sulfur in fossil fuel is converted into SO_2_ during combustion, and fossil fuel combustion accounts for the greatest proportion of anthropogenic releases globally (Brauer et al. 2002). In Poland, energy production is based on the combustion of hard and brown coal. Therefore, the outdoor 24-h concentrations of SO_2_ in Dąbrowa Górnicza and Złoty Potok (Poland) were higher than in Belgium. On the other hand, in Turkey, the mineral industry and the oil and brown coal energy grid are much better developed than in Poland. Therefore, the outdoor 24-h concentrations of SO_2_ in the urbanized region of Turkey were much higher than in the Silesian region of Poland.

It could be noted, that in all the abovementioned cases, the outdoor 24-h concentrations of SO_2_ were significantly higher than the indoor ones. In Dąbrowa Górnicza, 24-h I/O ratios varied from 0.39 to 0.98 with the median of 0.67. The I/O ratios of 24-h indoor and outdoor concentrations of SO_2_ in Złoty Potok varied from 0.39 to 2.52 with the median value of 1.28 (Table 3). Similar or lower values of I/O ratio were observed in previously cited studies in Belgium and Turkey (Stranger et al. 2008; Bozkurt et al. 2015), and also in urban and suburban schools in Brazil (Godoi et al. 2013) and in Korea (Kim et al. 2011). This may suggest that if there are no internal sources of SO_2_, which is quite typical of kindergarten rooms, the indoor and outdoor concentrations do not have to be at comparable levels. It seems that regardless of the degree of atmospheric pollution caused by SO_2_, the concentrations of SO_2_ in non-ventilated classrooms without additional internal sources are generally low or lower than in the atmospheric air. It is probably caused by photochemical transformation of SO_2_ in the atmospheric air, and consequently, by migration of products such as sulfuric acid and sulfur aerosol (e.g., (NH_4_)_2_SO_2_) into the rooms. SO_2_ is chemically short-lived in virtually all types of atmospheres (Hu et al. 2013). In addition, different thermodynamic conditions in the room and in the atmosphere cause that the intensity and direction as well as the complexity of the SO_2_ conversion in both environments are incomparable. However, positive (but not statistically significant) correlations between the indoor and outdoor 24-h concentrations of SO_2_ were confirmed only in the kindergarten in Dąbrowa Górnicza (Table 4). In contrast, values of I/O higher than in Dąbrowa Górnicza and the lack of correlation between indoor and outdoor 24-h concentrations of SO_2_ indicated the existence of internal sources of SO_2_ in the kindergarten in Złoty Potok (Table 5). In this kindergarten, a kitchen coal stove was used, which caused the increase of the indoor concentrations of SO_2_. Therefore, it can be concluded that the indoor air quality in Złoty Potok mainly depended on the intensity of coal or wood combustion in the kitchen stove, rather than on the atmospheric levels of SO_2_.

**Table 3:** The ratio of I/O for each parameter depending on the studied area

Pollutant	Dąbrowa Górnicza			Złoty Potok		
	Mean ± SE	Median	Range	Mean ± SE	Median	Range
SO_2_	0.67 ± 0.06	0.67	0.39–0.98	1.30 ± 0.25	1.28	0.39–2.52
NO_2_	0.28 ± 0.05	0.26	0.14–0.49	0.79 ± 0.12	0.62	0.47–1.28
PM2.5	0.87 ± 0.06	0.86	0.71–1.10	1.04 ± 0.15	1.17	0.52–1.50
BaP	0.90 ± 0.07	0.89	0.67–1.20	1.69 ± 0.41	1.66	0.58–3.76
Σ15 PAHs^a^	0.71 ± 0.04	0.72	0.55–0.85	0.99 ± 0.21	0.80	0.43–2.08
TA98 − S9	0.46 ± 0.04	0.43	0.41–0.58	0.49 ± 0.05	0.48	0.38–0.63
TA98 + S9	0.56 ± 0.10	0.49	0.43–0.84	0.65 ± 0.09	0.65	0.44–0.86
YG1024 − S9	0.24 ± 0.04	0.22	0.18–0.34	0.48 ± 0.08	0.50	0.26–0.65
YG1024 + S9	0.58 ± 0.12	0.54	0.35–0.88	0.27 ± 0.05	0.27	0.15–0.39

^a^Naphthalene, acenaphthene, fluorine, phenanthrene, anthracene, fluoranthene, pyrene, benz(a)anthracene, chrysene, benzo(b)fluoranthene, benzo(k)fluoranthene, benzo(a)pyrene, benzo(g,h,i)perylene, dibenz(a,h)anthracene, and indeno(1,2,3-c,d)pyrene

**Table 4:** Spearman correlation between indoor and outdoor air pollutants in Dąbrowa Górnicza

Dąbrowa Górnicza	Outdoor				
Indoor	SO_2_	NO_2_	PM2.5	BaP	Σ15 PAHs^a^
SO_2_	0.611				
NO_2_		− 0.357			
PM2.5			0.643	0.178	0.321
BaP			0.821^b^	0.893^b^	0.964^b^
Σ15 PAHs^a^			0.786^b^	0.821^b^	0.928^b^

^a^Naphthalene, acenaphthene, fluorine, phenanthrene, anthracene, fluoranthene, pyrene, benz(a)anthracene, chrysene, benzo(b)fluoranthene, benzo(k)fluoranthene, benzo(a)pyrene, benzo(g,h,i)perylene, dibenz(a,h)anthracene, and indeno(1,2,3-c,d)pyrene

^b^Correlation is significant at the level *p* ≤ 0.05

**Table 5:** Spearman correlation between indoor and outdoor air pollutants in Złoty Potok

Złoty Potok	Outdoor				
Indoor	SO_2_	NO_2_	PM2.5	BaP	Σ15 PAHs^a^
SO_2_	− 0.120				
NO_2_		0.430			
PM2.5			− 0.321	0.143	0.143
BaP			0.571	0.357	0.357
Σ15 PAHs^a^			0.071	0.214	0.,214

^a^Naphthalene, acenaphthene, fluorine, phenanthrene, anthracene, fluoranthene, pyrene, benz(a)anthracene, chrysene, benzo(b)fluoranthene, benzo(k)fluoranthene, benzo(a)pyrene, benzo(g,h,i)perylene, dibenz(a,h)anthracene, indeno(1,2,3-c,d)pyrene

### Nitrogen dioxide

The indoor 24-h concentrations of NO_2_ ranged from 6.8 to 9.8 μg/m^3^ with the median of 8.1 μg/m^3^ for Dąbrowa Górnicza and from 4.2 to 13.5 μg/m^3^ with the median of 7.8 μg/m^3^ for Złoty Potok. The outdoor 24-h concentrations of NO_2_ ranged from 19.0 to 55.0 μg/m^3^ with the median value of 34.5 μg/m^3^ for Dąbrowa Górnicza; for Złoty Potok, they ranged from 8.0 to 17.0 μg/m^3^ with the median value of 10.0 μg/m^3^ (Table 2). Significantly higher concentrations of NO_2_ were found in the atmospheric air of Dąbrowa Górnicza than in Złoty Potok (*p* = 0.001).

Similar average outdoor concentrations of NO_2_ as in Złoty Potok were recorded in the suburban areas of Stockholm (Wichmann et al. 2010). The previous study conducted in ten kindergartens, six schools in Sweden, and two schools in Portugal showed slightly higher concentrations of NO_2_ than in the Silesian kindergartens (Wichmann et al. 2010; Pegas et al. 2012). In Spain and Turkey, higher concentrations of NO_2_ were observed both in schools and in the outdoor air (Rivas et al. 2014; Demirel et al. 2014; Bozkurt et al. 2015).

The concentrations of NO_2_ are more spatially varied than the concentrations of SO_2_. The levels of NO_2_ depended on emissions from various sources. Primarily, from the rapidly changing in space and time, road traffic emission as well as thermodynamic and meteorological conditions in the air of the studied area had an influence on the speed, intensity, and direction of photochemical reactions in the atmosphere (Carslaw 2005; Beevers et al. 2012). High variability of NO_2_ concentrations in Polish areas in comparison to previously mentioned countries could be considered as an effect of the road traffic intensity in big cities, resulting in higher atmospheric concentrations of NO_x_.

Statistically higher concentrations of NO_2_ in Dąbrowa Górnicza in comparison to Złoty Potok were definitely associated with differences in the road traffic emission. Dąbrowa Górnicza was characterized by higher intensity of road traffic than Złoty Potok localized in a rural region. What is more, dispersed and low-rise housing typical of Złoty Potok encouraged high intensity of NO_2_ transformations and consequently a reduction of the NO_2_ levels in the outdoor air (Seinfeld and Pandis 2006).

In this study, NO_2_ concentrations in the atmospheric air were significantly higher than in the air of kindergartens both in Dąbrowa Górnicza (*p* = 0.000) and Złoty Potok (*p* = 0.016)—Table 1. The ratio of the 24-h indoor concentrations of NO_2_ to 24-h outdoor concentrations in Dąbrowa Górnicza varied from 0.14 to 0.49 with the median value of 0.26. The 24-h I/O ratio of NO_2_ in Złoty Potok varied from 0.47 to 1.28 with the median value of 0.62 (Table 3). In Dąbrowa Górnicza, the values of I/O ratio and weak correlation between 24-h indoor and outdoor concentrations of NO_2_ indicated that the pollutant came mainly from outdoor sources (Tables 3 and 4). Very similar results were observed during winter in elementary schools located in the central and suburban areas of Aveiro, Portugal (0.72 and 0.68), schools in Barcelona, Spain (0.63), and a school in Kocaeli, Turkey (0.86) (Pegas et al. 2012; Rivas et al. 2014; Bozkurt et al. 2015).

Similarly to indoor SO_2_ concentrations, also the indoor levels of NO_2_ in places where there are no significant internal sources of NO_2_ are very likely to be lower than in the atmospheric air, mainly due to different transformations of NO_x_ compounds in indoor and outdoor environments. A different situation was observed in Złoty Potok, where a kitchen stove fired with coal and a gas stove was used. In this case, I/O ratio for NO_2_ was greater than 1 (Table 3), but a positive correlation (*r* = 0.430, *p* > 0.05) between the indoor and outdoor 24-h concentrations of NO_2_ was also detected (Table 5). The I/O ratio calculated for NO_2_ indicated the influence of internal sources of NO_2_ in the kindergarten in Złoty Potok, whereas the relationship between the indoor and outdoor 24-h concentrations of NO_2_ can be considered to be the effect of infiltration of atmospheric NO_2_. Generally, it was observed that the impact of the kitchen stove emission in the kindergarten in Złoty Potok was much stronger in the case of SO_2_ indoor concentrations than in the case of NO_2_.

### Fine particulate matter PM2.5

Twenty-four-hour concentrations of PM2.5 inside kindergartens ranged from 18.5 to 42.4 μg/m^3^ with the median of 25.1 μg/m^3^ in Dąbrowa Górnicza and from 20.0 to 41.9 μg/m^3^ with the median value of 36.1 μg/m^3^ in Złoty Potok. Twenty-four-hour ambient concentrations of PM2.5 in the atmospheric air ranged from 22.8 to 45.0 μg/m^3^ (median 32.5 μg/m^3^) in Dąbrowa Górnicza and from 16.0 to 69.5 μg/m^3^ (median 30.6 μg/m^3^) in Złoty Potok (Table 2).

During winter 2013/2014 in Silesia the indoor concentrations of PM2.5 in nursery schools were even higher (94.1 μg/m^3^ in the urban site and 66.7 μg/m^3^ in the rural site). In the same period, the lowest outdoor concentration of PM2.5 (21.88 μg/m^3^) was detected in the air of the nursery school playgrounds located in the urban traffic area, whereas the highest level (88.30 μg/m^3^) was observed at the playground located in the rural site (Mainka and Zajusz-Zubek 2015).

The mean concentration of PM2.5 in the atmospheric air in both sites was relatively high; it exceeded 30 μg/m^3^ (Table 2). The PM2.5 measurement results obtained in this study come from a short sampling period, and, therefore, comparing the mean concentrations obtained for such a period with the annual permissible level of PM2.5 (25 μg/m^3^, Table 1) is not adequate. However, based on the comparison of these results with the values obtained earlier in other cities of the same region, it can be assumed that the annual average for PM2.5 in the cities of southern Poland, including Dąbrowa Górnicza and Złoty Potok are very likely to be exceeded.

In Poland, much higher levels of particulate pollutants are characteristics for heating periods as a result of local emission from house heating—coal and wood combustion in domestic stoves and increased production in heating or/and power plants. This was confirmed by previous studies of PM1, PM2.5, and PM10 conducted in Złoty Potok and cities such as Katowice and Zabrze, located in the vicinity of Dąbrowa Górnicza (Rogula-Kozłowska et al. 2013a, b, 2014).

Detected in Polish cities—especially in the southern region of the country—average annual PM fine concentrations are practically the highest in Europe (Błaszczak et al. 2016). The high average annual concentrations of PM2.5 correspond well with very high values of 24-h concentrations, which are recorded in the southern part of Poland during the winter/heating season (Rogula-Kozłowska et al. 2014; Błaszczak et al. 2016). In Poland, in contrast to other European cities, very high concentrations are observed in areas outside the cities, with poorly developed heating network and attempts to reduce heating costs make residents heat their houses by burning in domestic furnaces, not only coal and wood but also various types of waste. In the results discussed in this paper, despite the fact that the research was conducted in spring, the heating season continued and slightly higher PM2.5 concentrations were recorded in the village of Złoty Potok than in the large, industrialized city of Dąbrowa Górnicza (Table 2).

The 24-h indoor concentrations of PM2.5 in Dąbrowa Górnicza were a little higher than outdoors, and the 24-h I/O ratios varied from 0.71 to 1.10 with the median value of 0.86. The ratios of 24-h indoor concentrations of PM2.5 to 24-h outdoor concentrations of PM2.5 in Złoty Potok varied from 0.52 to 1.50 with the median of 1.17 (Table 3). There were no significant differences between the indoor and outdoor 24-h concentrations of PM2.5 both in Dąbrowa Górnicza and Złoty Potok (Table 2). However, the average concentration of PM2.5 in both kindergartens was slightly lower than the average concentration of PM2.5 in the atmospheric air. Generally, fine particulate fraction freely migrates into the rooms even when the windows are closed, so PM2.5 indoor and outdoor concentrations are usually similar or slightly higher inside the poorly ventilated rooms, where there are no internal PM sources. Usually, the I/O ratio in well-ventilated rooms or with frequent airing is approx. 1.

The previously carried out studies showed that the average value of I/O ratio for PM2.5 in elementary schools in Prague was 0.94, in schools in Stockholm was 0.84, and 0.82 in three primary schools in Rome (Braniš et al. 2009; Wichmann et al. 2010; Tofful and Perrino 2015). Also, in the studies conducted in lecture rooms located in Polish cities, in Gliwice (Upper Silesia) and in Warsaw (central part of Poland) the indoor and outdoor concentrations of PM1 were almost equal or slightly lower (Majewski et al. 2016; Rogula-Kozłowska et al. 2017). The average I/O ratio for PM2.5 below 1 was found in two nursery schools in the urban and rural area in the Upper Silesia (0.97 and 0.92) in rooms occupied by younger children (Mainka and Zajusz-Zubek 2015).

There was a significant correlation between indoor and outdoor 24-h concentrations of PM2.5 (*r* = 0.643, *p* > 0.050), indicating that the indoor PM2.5 in the kindergarten in Dąbrowa Górnicza was impacted mainly by the outdoor PM2.5, whereas in the kindergarten in Złoty Potok, such a relationship between the indoor and outdoor 24-h concentrations of PM2.5 was not observed (Tables 4 and 5). Moreover, in Złoty Potok, the I/O ratio was above 1 for 4 out of 7 days. As in the case of gaseous pollutants, also in the case of PM2.5, the impact of the emission from the kitchen coal stove on the quality of indoor air in the kindergarten located in Złoty Potok was observed. In the case of PM2.5, this effect is quite clear. Of course, it cannot be excluded that the worse air quality in Złoty Potok than in Dąbrowa Górnicza might be caused by the worse air exchange in the rural kindergarten.

### PM2.5-bound PAHs

The indoor 24-h concentrations of the main representative of the group of PAHs-BaP ranged from 1.2 to 7.5 ng/m^3^ with the median concentration of 3.6 ng/m^3^ for Dąbrowa Górnicza and from 2.7 to 12.8 with the median value of 3.1 ng/m^3^ for Złoty Potok. The average outdoor BaP concentration was 4.0 ng/m^3^ for Dąbrowa Górnicza and 3.1 ng/m^3^ for Złoty Potok (Table 2). The 24-h BaP concentrations in all PM2.5 samples were equal or higher than 1 ng/m^3^ (annual permissible level of BaP; Directive 2004). The previously conducted studies showed that 24-h concentrations of BaP during winter/heating season in these areas were higher than those observed during spring season (Rogula-Kozłowska et al. 2013a, b). These data suggest that the annual permissible level of BaP in the south of Poland probably will not be kept. BaP similar to SO_2_ and particulate fractions originated mainly from the combustion of fossil fuels in energy production processes in both domestic stoves and power plants. What is more, in Poland more than 90% of electricity is produced in coal-fired power plants, and for this reason, the atmospheric concentrations of BaP are very high. BaP levels are more comparable with data observed in big Asian agglomerations than in the European cities (Rogula-Kozłowska 2015).

On the other hand, in the carried out study the sum of 15 PM2.5-bound PAHs was lower (Table 2) than the summarized PAHs concentrations in relation to the levels observed in the European areas (Rogula-Kozłowska 2015). The 24-h indoor concentrations of the sum of 15 PM2.5-bound PAHs (Σ15PAHs) ranged from 14.6 to 72.9 ng/m^3^ with the median value of 36.1 ng/m^3^ for Dąbrowa Górnicza and from 25.0 to 89.5 ng/m^3^ with the median of 31.4 ng/m^3^ for Złoty Potok. The average outdoor concentrations of Σ15PAHs for Dąbrowa Górnicza and Złoty Potok were equal to 52.9 and 39.5 ng/m^3^ (Table 2). Beyond the heating season, the occurrence and concentrations of the majority of compounds of the PAH group (except for some, including BaP) are connected with transport emissions. Taking into consideration, the fact that the communication network in the areas of the conducted studies is less developed, and the number of cars is much smaller than in many other heavily urbanized regions, such a situation may be regarded as natural. A similar relationship was also observed for NO_2_, which is connected with road traffic sources.

The BaP concentrations in indoor and outdoor microenvironments were higher in the rural area (Złoty Potok) in comparison to the urbanized city (Dąbrowa Górnicza). A different situation was observed in the case of Σ15PAHs, which are influenced not only by emissions from fossil fuel combustion, but mainly from transport sources. Therefore, during spring season in Dąbrowa Górnicza, higher concentrations of Σ15PAHs were detected in comparison to Złoty Potok. In both areas, the differences between the indoor and outdoor 24-h concentrations of BaP and Σ15PAHs were not statistically significant, and no differences between the examined areas were observed, both in the case of the indoor and outdoor air. Incidentally, high 24-h concentration of BaP was detected in samples collected in the kindergarten in Złoty Potok. The 24-h I/O ratios of indoor concentrations of BaP in Dąbrowa Górnicza varied from 0.67 to 1.20 with the median of 0.89. Indoor 24-h concentrations of BaP in Złoty Potok were higher than the outdoor ones (but not significant: *p* = 0.061), and the 24-h values of the I/O ratio varied from 0.58 to 3.76 with the median value of 1.66 (Table 3). Twenty-four-hour I/O ratios for Σ15 PAHs in Dąbrowa Górnicza varied from 0.55 to 0.85 with the median value of 0.72. Twenty-four-hour I/O ratios for Σ15PAHs in Złoty Potok varied from 0.43 to 2.08 with the median of 0.80 (Table 3). Twenty-four-hour indoor concentrations of both BaP and Σ15PAHs showed strong and positive correlation with the 24-h outdoor concentrations of BaP and Σ15PAHs (*r* = 0.893, *p* ≤ 0.05 for BaP and *r* = 0.928, *p* ≤ 0.05 for Σ15PAHs) in Dąbrowa Górnicza, which suggested that PM2.5-bound BaP and Σ15PAHs in the urban kindergarten came mainly from the outdoor environment. In the kindergarten in Złoty Potok, the lack of statistically significant correlations between the 24-h indoor and outdoor concentrations of both BaP and Σ15PAHs confirmed the earlier assumption of the existence of internal sources of air pollutants (Table 5). Like in the case of PM and SO_2_, also for PAHs, and in particular for BaP, the burning of wood and coal in the kitchen stove in the Złoty Potok kindergarten, apart from infiltration of the atmospheric air, was an important internal source of emission.

It should be pointed out that no safe threshold can be determined, and all indoor exposures to PAHs are considered relevant to health. Unit risk for lung cancer for PAH mixtures is estimated at 8.7 × 10^−5^ per nanogram per cubic meter of BaP. This is the guideline for PAHs in the indoor air. The corresponding concentrations for lifetime exposure to BaP producing excess lifetime cancer risks of 1/10,000, 1/100,000, and 1/1,000,000 are approximately 1.2, 0.12, and 0.012 ng/m^3^, respectively (WHO 2010). In this study, based on short-term measurements of the indoor 24-h concentrations of BaP in both locations, the cancer risk was calculated using the US EPA approach (US EPA 2009). The exposure parameters used for risk calculation were presented in the Supplementary Material (SM3). The cancer risks resulting from the children’s exposure to air pollutants in Dąbrowa Górnicza and Złoty Potok amounted to 2.2 × 10^−5^ and 3.4 × 10^−5^, respectively. It means that the excess lifetime cancer risks were one order of magnitude above the level of 10^−6^, the most often considered as acceptable. High levels of this pollutant in the indoor air may, in consequence, cause specific long-term health effects, i.e., lung cancer.

### *Salmonella*/microsome assay

Table 2 shows the mutagenicity data for the organic extracts from indoor and outdoor PM2.5 (rev/m^3^). Cytotoxic effects were not detected for any of the analyzed samples.

#### TA98 strain

The mutagenic effect of 24-h indoor samples of PM2.5 tested using the TA98 strain without metabolic activation ranged from 21.0 to 21.9 rev/m^3^ with the median of 21.2 rev/m^3^ in Dąbrowa Górnicza and from 18.6 to 23.1 rev/m^3^ with the median of 19.6 rev/m^3^ in Złoty Potok. In the variant with metabolic activation, the 24-h mutagenic effect ranged from 27.0 to 28.4 rev/m^3^ with the median of 28.3 rev/m^3^ in Dąbrowa Górnicza and from 27.8 to 30.4 rev/m^3^ with the median of 27.8 rev/m^3^ in Złoty Potok (Table 2).

In the available bioscience databases, only two publications concerning the mutagenicity of particulate matter in the indoor environment were found. Significantly higher mutagenic effect of PM2.5 detected using the TA98 strain with metabolic activation were found in cars, restaurants, offices, or living and sleeping rooms of cigarette smokers in China (60.4–595.5 rev/m^3^) (Zhou et al. 2000).

The mutagenic effect of 24-h outdoor samples of PM2.5 detected using the TA98 strain without metabolic activation (TA98-S9, direct mutagenicity) ranged from 36.6 to 51.8 rev/m^3^ with the median of 50.3 rev/m^3^ in Dąbrowa Górnicza and from 32.6 to 60.8 rev/m^3^ with the median of 39.5 rev/m^3^ in Złoty Potok. In the variant with metabolic activation (TA98 + S9, indirect mutagenicity), the mutagenic effect of 24-h PM2.5 samples ranged from 33.1 to 66.6 rev/m^3^ with the median of 56.8 rev/m^3^ for Dąbrowa Górnicza and from 33.5 to 62.7 rev/m^3^ with the median of 45.4 rev/m^3^ for Złoty Potok (Table 2).

Direct mutagenic effect of PM10 samples collected in winter (1999/2000) at 21 measuring stations of the Regional Sanitary-Epidemiological Station in Katowice tested using the TA98 strain (49.0 rev/m^3^) was similar to the value obtained in our study, whereas the indirect effect appeared to be twice as high (99.0 rev/m^3^). Then, the average concentration of PM10-bound BaP was much higher (28.8 ng/m^3^) than in the present study—Table 2 (Mielżyńska et al. 2002). A similar mutagenic effect of PM10 tested using the TA98 strain was found in Częstochowa and Dąbrowa Górnicza, without (50 and 65 rev/m^3^, respectively) and after metabolic activation (125 and 151 rev/m^3^, respectively) (Kozłowska et al. 2007).

A similar mutagenic effect of PM10, both without (39.5 rev/m^3^) and with the addition of S9 (35.8 rev/m^3^), was observed in winter in Belgium (Du Four et al. 2004). A lower mutagenic effect of PM10 detected using TA98-S9 (< 13.0 rev/m^3^) and TA98 + S9 (< 25.0 rev/m^3^) was found in the study conducted during winter (2000/2001) in Teplice and Prague (Binková et al. 2003).

#### YG1024 strain

Mutagenic effect of 24-h indoor samples of PM2.5 detected using the YG1024 strain without metabolic activation ranged from 53.6 to 79.0 rev/m^3^ with the median of 56.7 rev/m^3^ in Dąbrowa Górnicza and from 53.7 to 63.8 rev/m^3^ with the median of 58.4 rev/m^3^ in Złoty Potok. However, in the variant with metabolic activation, the 24-h mutagenic effect ranged from 68.9 to 146.7 rev/m^3^ with the median of 85.4 rev/m^3^ in Dąbrowa Górnicza and from 72.9 to 80.4 rev/m^3^ with the median of 73.2 rev/m^3^ in Złoty Potok (Table 2).

As opposed to the present study, in 22 houses in Tokyo, the direct mutagenic effect of PM2.5 tested using the YG1024 strain was much higher than the indirect effect tested with the same strain (Takagi et al. 2002).

The mutagenic effect of 24-h outdoor samples of PM2.5 detected using the YG1024 strain without metabolic activation (YG1024-S9, direct mutagenicity) ranged from 93.1 to 175.5 rev/m^3^ with the median of 146.7 rev/m^3^ in Dąbrowa Górnicza and from 89.6 to 204.2 rev/m^3^ with the median of 121.9 rev/m^3^ in Złoty Potok. In the variant with metabolic activation (YG1024 + S9, indirect mutagenicity), the mutagenic effect of 24-h PM2.5 samples ranged from 114.0 to 246.4 rev/m^3^ with the median of 192.6 rev/m^3^ in Dąbrowa Górnicza and from 105.2 to 270.9 rev/m^3^ with the median of 150.6 rev/m^3^ in Złoty Potok (Table 2).

A definitely lower mutagenic effect of outdoor particulate matter tested using the YG1024 strain was detected in Saitama (Japan), in both variants (− S9: 95.6 rev/m^3^; + S9: 32.9 rev/m^3^) (Kawanaka et al. 2004). Very low biological activity of outdoor PM2.5 for the YG1024 strain, collected during winter in Rio Grande do Sul (Brazil), was found in the variant without metabolic activation (9.5 rev/m^3^), as well as after its application (2.1 rev/m^3^) (da Silva et al. 2015).

There were no statistically significant differences between the mutagenic effect of indoor and outdoor PM2.5 in Dąbrowa Górnicza and Złoty Potok, regardless of the applied strain (TA98, YG1024) and the variant of activation (± S9). The mutagenic effect of 24-h indoor PM2.5 samples tested using both TA98 and YG1024 strains was twice as low as that of the 24-h outdoor PM2.5 samples.

Frameshift mutagenicity tested using TA98 and YG2024 strains in airborne particulate matter extracts, from indoor and outdoor PM2.5 samples collected in urban (Dąbrowa Górnicza) and rural (Złoty Potok) sites, was observed during the spring period (March to May). The results showed that the mutagenicity tested using the YG1024 strain was much higher than in the case of TA98 in almost all samples, both indoors and outdoors. The YG1024 strain is extremely sensitive to aromatic amines, hydroxylamines, and nitro-PAHs, as was previously described. Furthermore, in almost all samples, mutagenicity with S9 fraction was not significantly higher than without S9 fraction. These results indicate that indirect-acting mutagenic compounds such as aromatic amines may be responsible for this effect (da Silva et al. 2015). Unsubstituted PAH compounds occurring in PM extracts belong to promutagens, and their mutagenic properties are detected in *Salmonella* assay in the presence of S9. In the previously conducted Polish studies, the increase of mutagenic activity tested by TA98, YG1021, and YG1024 strains after addiction of S9 fraction to PM10 samples collected during winter season was observed. In summer, indirect mutagenicity was similar or lower in comparison to the variant without metabolic activation (Bubak 1998).

However, unsubstituted PAHs cannot account for the total observed mutagenicity. Neither BaP nor the sum of 15 selected PAHs could serve as a good exhaustive air quality indicator. The presence of mutagenic pollutants other than the abovementioned PAHs, which was not monitored in this work, needs further investigations. The studied pollutants may play a role in determining the *Salmonella* response, but they cannot explain the mutagenic phenomenon in full. In conclusion, it is very likely that PAHs and their derivatives contributed to the detected airborne mutagenicity. However, the commonly analyzed PAHs accounted for less than 5% of the total detected mutagenicity, because the air contains many uncharacterized genotoxicants (Ducatti and Vargas 2003). Moreover, the mutagenic effect of PAH mixtures in environmental samples is lower than in the case of an individual compound of PAHs. Also, the origin of PAHs, the sampling season, PM composition, and the applied *Salmonella* strain had an influence on the obtained results. However, these are detailed chemical analyses or fractionation that can confirm which class of genotoxic chemicals is responsible for mutagenicity observed in the bacterial test.

## Summary and conclusions

The indoor air quality parameters (concentrations of SO_2_, NO_2_, PM2.5, BaP, sum of 15 PM2.5-bound PAHs, and mutagenicity of PM2.5) were characterized in naturally ventilated kindergartens in urban (Dąbrowa Górnicza) and rural (Złoty Potok) areas in Silesia, Poland during spring season. The assessment of the indoor air quality was made taking into consideration the outdoor concentrations of the studied pollutants.

In Dąbrowa Górnicza, statistically lower 24-h concentrations of SO_2_ and NO_2_ were observed in the indoor air, whereas in Złoty Potok, such dependence was observed only for NO_2_. Nevertheless, the results obtained in the current study showed that the air in the analyzed kindergartens was quite safe with respect to SO_2_ and NO_2_, as their concentrations were well below the defined harmful thresholds.

There were no statistically significant differences for 24-h concentrations of PM2.5, BaP, and the sum of 15 PM2.5-bound PAHs in kindergartens and the atmospheric air in Dąbrowa Górnicza and Złoty Potok. The level of direct and indirect mutagenicity of indoor and outdoor PM2.5 samples in the case of the two analyzed strains was comparable for the two studied areas. The mutagenic effect of indoor PM2.5, detected using TA98 and YG1024 strains was twice as low as in the case of outdoor PM2.5. In PM2.5 samples from both kindergartens, higher mutagenicity was observed for the strain YG1024 with metabolic activation in comparison to the variant without activation. These results observed for the YG strain suggested the presence of aromatic amines, hydroxylamines, and also nitrogenated compounds. The presence of co-pollutants in the mixture of airborne particles resulting from seasonal variation and emission sources have an influence on the total mutagenic activity tested by TA98 and YG1024 strains. It suggests that the monitoring of atmospheric pollution should be complemented with the studies on mutagenicity of suspended particulates with the *Salmonella* assay.

The obtained results indicated that PM2.5 and the sum of 15 PM2.5-bound PAHs, as well as *Salmonella* mutagenicity of PM2.5, occurring both in the air inside and outside kindergartens in Silesia, are an important source of children’s exposure to genotoxic agents.

In the kindergarten located in Dąbrowa Górnicza, gaseous pollutants and PM2.5, BaP, and the sum of 15 PM2.5-bound PAHs mainly came from the atmospheric air. In the kindergarten in Złoty Potok, an internal source of SO_2_, PM2.5, and BaP existed. It was probably the emission from gas or/and coal stoves, which were used to prepare meals for children. In contrast to the obtained concentrations of PM2.5 and BaP, the mutagenic effects (strains TA98, YG1024) of indoor PM2.5 samples were lower than in the case of outdoor PM2.5 samples. This fact indicates that the total mutagenicity of PM2.5 fraction does not correspond to the results obtained in the chemical analysis.

Children spend an important part of their time in enclosed space (homes, kindergartens, and schools); they are also more sensitive to air pollution than adults. We believe that the results of the current study will point out to the need for implementing a strategy to control air quality in places where children reside. Undoubtedly, no kitchen coal stoves should be used in kindergartens and schools, and the existing ones should be replaced by electrical or gas stoves. In the newly built kindergartens or schools, mechanical ventilation with an air purification system should be installed. In old buildings, frequent measurements of the efficiency of the ventilation systems should be carried out, and more attention should be paid to the appropriate and sufficient exchange of air in rooms where children are staying, e.g., frequent airing of rooms. Development and implementation of guidelines for harmful indoor air pollutants would also be recommended.

## Electronic supplementary material

ESM 1 (DOC 9395 kb)
