# Supplementary material for: Indoor air quality in urban and rural kindergartens: short-term studies in Silesia, Poland
Source: Air Qual Atmos Health. 2017 Aug 17;10(10):1207–20. doi: 10.1007/s11869-017-0505-9 (PMC5741794; doi:10.1007/s11869-017-0505-9)
Supplement: Supplementary file 1 — (DOC 9395 kb) [file 11869_2017_505_MOESM1_ESM.doc]

**Supplementary material for**

**Indoor air quality in urban and rural kindergartens: short-term studies in Silesia, Poland**

Ewa Błaszczyk^1*^, Wioletta Rogula-Kozłowska^2^, Krzysztof Klejnowski^2^, Piotr Kubiesa^1^, Izabela Fulara^3^, Danuta Mielżyńska-Švach^1,4^

^1^ Environmental Toxicology Group, Institute for Ecology of Industrial Areas, Katowice, Poland

^2^ Department of Air Protection, Institute of Environmental Engineering, Polish Academy of Science, Zabrze, Poland

^3^ Central Laboratory, Institute for Ecology of Industrial Areas, Katowice, Poland

^4^ Nursing Institute, Witold Pilecki State School of Higher Education, Oświęcim, Poland

Corresponding author: Ewa Błaszczyk (Ph.D.), e-mail: e.blaszczyk@ietu.pl

**
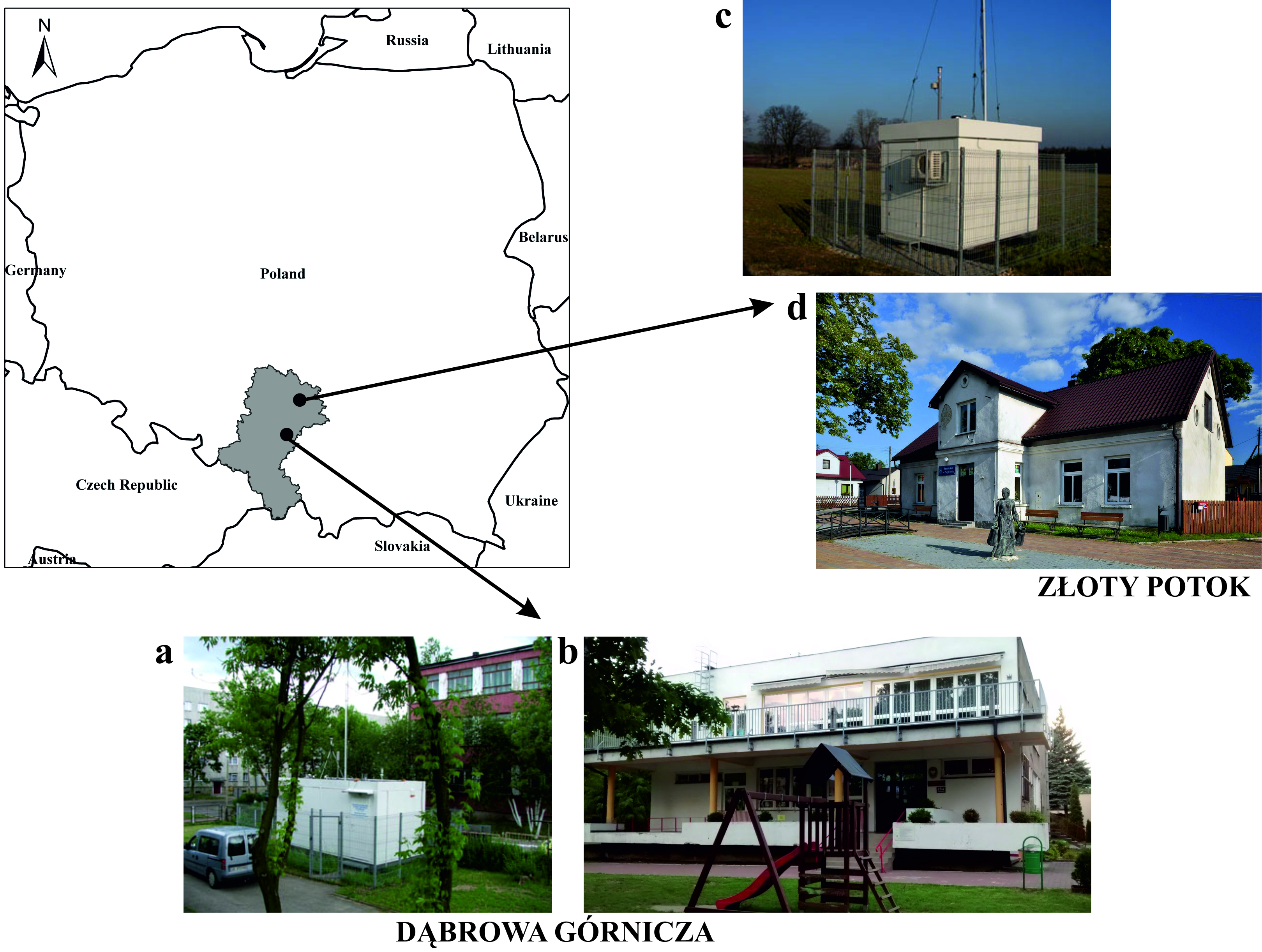
**

**SM 1.** Localization of sampling sites in Silesian voivodship; photographs: outdoor air monitoring station (a), kindergarten in Dąbrowa Górnicza (b), outdoor air monitoring station (c) and kindergarten in Złoty Potok (d)

**SM 2. Detailed characteristics of kindergarten buildings**

| **Parameter** | **Kindergarten** | |
| --- | --- | --- |
|  | **Dąbrowa Górnicza** | **Złoty Potok** |
| **Number of children (group)** | 125 children (6 groups) | 25 children (1 group) |
| **Heating system** | central heating from the municipal network | electric heating |
| **Window frame material** | plastic and wood | wood |
| **Kitchen stove type** | gas stove | gas and coal stove |
| **Floor type** | PCV flooring | carpets and laminate wood floor |
| **Ventilation system** | gravitational | gravitational |

**SM3.** Parameters of exposure concentration (EC) and inhalation unit risk (IUR) used to calculation of inhalation cancer risk for children exposure to BaP in kindergartens

| **Parameter** | **Abbreviation** | | **Value** |
| --- | --- | --- | --- |
| BaP concentration in air | CA | | 3.7 ng/m^3^ for Dąbrowa Górnicza  5.7 ng/m^3^ for Złoty Potok |
| Exposure time | ET | | 5 hours/day |
| Exposure frequency | EF | | 120 days/year* |
| Exposure duration | ED | | 3 years |
| Averaging time | AT | | 26280 hours |
| Inhalation Unit Risk | IUR | | 8.7×10^-5^ |
| **Equations**** | | | |
| **Exposure concentration (EC):**  EC = (CA × ET × EF × ED) / AT | | **Cancer risk:**  Risk = IUR × EC | |

* - concerned only heating season;

** - according to US EPA (2009) Risk assessment guidance for superfund, vol. I: Human health evaluation manual, part F: Supplemental guidance for inhalation risk assessment. Environmental Protection Agency, Washington, D.C.;
